# Utility of Serum Homocysteine and Lipoprotein(a) in Acute Coronary Syndrome: A Cross-Sectional Study From Western India

**DOI:** 10.7759/cureus.111472

**Published:** 2026-06-25

**Authors:** Bhupender Arya, Pyrus Bhellum, Priyanka Agnihotri, Shyam Lal Mathur

**Affiliations:** 1 Department of General Medicine, All India Institute of Medical Sciences, Jodhpur, IND; 2 Department of Pathology, Sawai Man Singh Medical College, Jaipur, IND; 3 Department of Internal Medicine, Dr. Sampurnanand Medical College, Jodhpur, IND

**Keywords:** acute coronary syndrome, cardiovascular risk factors., hyperhomocysteinemia, serum homocysteine, serum lipoprotein (a), young cad

## Abstract

Background: Acute coronary syndrome (ACS) remains a major cause of cardiovascular morbidity and mortality in India, with disease onset occurring at younger ages than in Western populations. Serum homocysteine and lipoprotein(a) Lp(a) are recognized non-conventional risk factors whose interrelationship and age-specific relevance in Indian patients with ACS remain poorly defined.

Methods: This cross-sectional study was undertaken at Dr. Sampurnanand Medical College and Mathura Das Mathur Hospital, Jodhpur, between January and November 2023. Seventy-five consecutive ACS patients (ST-segment elevation myocardial infarction (STEMI), non-ST-segment elevation myocardial infarction (NSTEMI), and unstable angina) were enrolled. Serum homocysteine and Lp(a) were measured by immunoturbidimetry, with Lp(a) ≥30 mg/dL defined as elevated. Receiver operating characteristic (ROC) curve analysis was performed to identify the optimal homocysteine threshold for predicting young-onset ACS.

Results: Younger patients (aged below 40 years) demonstrated significantly higher homocysteine levels than older counterparts (p=0.0009). Elevated Lp(a) was present in nearly half the cohort and was accompanied by significantly higher homocysteine concentrations (p<0.0001). Vitamin B12 deficiency was identified in over half of the patients, potentially contributing to hyperhomocysteinaemia.

Conclusion: Serum homocysteine is markedly elevated in young-onset ACS and correlates positively with Lp(a) levels. These findings suggest that screening for both biomarkers, alongside vitamin B12 assessment, may warrant consideration in the workup of ACS patients, particularly in the Indian population. However, given the cross-sectional, single-center design of the present study and the absence of outcome data, prospective multicenter studies are needed to validate these associations.

## Introduction

Acute coronary syndrome (ACS) is among the foremost contributors to cardiovascular morbidity and mortality worldwide, with the greatest burden borne by low- and middle-income countries. More than four-fifths of cardiovascular disease (CVD)-related deaths and disability arise in these settings, and the Indian population is particularly vulnerable, experiencing disease onset at younger ages and at higher frequencies compared with Western populations [1]. A comprehensive systematic review confirmed the high prevalence of coronary artery disease (CAD) risk factors in India, delayed hospital presentation, variable use of evidence-based treatments, and high in-hospital mortality among ACS patients [2].

Among the non-conventional risk factors implicated in CAD, particularly in young patients (<40 years), hyperhomocysteinaemia and elevated lipoprotein(a) (Lp(a)) have garnered significant attention, alongside dyslipidemia, smoking, impaired glucose tolerance, positive family history, and psychosocial stress [3]. Homocysteine is an intermediate in the methionine-to-cysteine pathway, requiring vitamin B12, folate, and methionine synthase for its remethylation [4]. Its accumulation promotes atherogenesis through low-density lipoprotein (LDL) thiolation, macrophage foam cell formation, and reactive oxygen species generation, causing direct injury to the endothelial, medial, and adventitial layers of the arterial wall and contributing to atherosclerosis, thrombosis, peripheral arterial disease, and aortic aneurysm [5-12]. Lp(a) and homocysteine are both classified as major emerging non-conventional cardiovascular risk factors alongside high-sensitivity C-reactive protein (hsCRP), interleukins, genetic markers, and environmental exposures such as obesity, physical inactivity, and depression [13]. Evidence further suggests that co-elevation of both biomarkers may confer a synergistic cardiovascular risk, particularly in women [14]. Notably, Asian and African populations demonstrate a stronger homocysteine-CAD association than their European counterparts, and this relationship has strengthened over time [15].

Both homocysteine and Lp(a) have been independently implicated in endothelial dysfunction, oxidative stress, and accelerated atherosclerosis; emerging evidence further suggests that hyperhomocysteinaemia may potentiate Lp(a)-mediated vascular injury through shared prothrombotic and pro-inflammatory pathways, raising the possibility of additive or synergistic cardiovascular risk. The present study was therefore undertaken to evaluate serum homocysteine levels across age groups in ACS patients and to examine their relationship with serum Lp(a), with the aim of generating clinically relevant regional data to inform preventive and therapeutic cardiology practice.

## Materials and methods

Study design and setting

This cross-sectional observational study was carried out at the Department of Medicine, Mathura Das Mathur Hospital and Mahatma Gandhi Hospital, Jodhpur, both affiliated with Dr. Sampurnanand Medical College, Jodhpur, Rajasthan, India, over a 10-month period spanning January to November 2023.

Study population

A total of 75 consecutive patients with a confirmed diagnosis of ACS were enrolled. ACS was diagnosed on the basis of clinical presentation, electrocardiographic changes (ST-elevation, ST-depression, or T-wave inversions), elevated cardiac biomarkers (serum troponin I/T), and echocardiographic findings, as appropriate. Patients were categorized into ST-segment elevation myocardial infarction (STEMI; n=35, 46.7%), non-ST-segment elevation myocardial infarction (NSTEMI; n=20, 26.7%), and unstable angina (n=20, 26.7%) according to standard definitions. Patients were further stratified into two age groups: <40 years (n=16, 21.3%) and ≥40 years (n=59, 78.7%).

Patients with known chronic kidney disease, hypothyroidism, hepatic dysfunction, active malignancy, pregnancy, or those already on vitamin B12/folate supplementation or homocysteine-lowering therapy were excluded, as these conditions independently alter homocysteine metabolism.

Sample collection and laboratory methods

After obtaining written informed consent from each participant, 2 mL of venous blood was collected in a plain vial under aseptic precautions. Samples were collected in the fasting state within 24 hours of admission and before initiation of relevant therapy; internal quality controls were performed contemporaneously with each analytical batch. Serum homocysteine and serum Lp(a) levels were measured by immunoturbidimetry. Serum vitamin B12 levels were also recorded. Lp(a) ≥30 mg/dL was considered elevated, in keeping with established cardiovascular risk thresholds. Hyperhomocysteinaemia was defined as serum homocysteine >15 µmol/L. Vitamin B12 deficiency was defined as serum B12 <200 pg/mL.

Ethical considerations

The study was conducted in accordance with the principles of the Declaration of Helsinki. Ethical approval was obtained from the Institutional Ethics Committee (IEC), Dr. Sampurnanand Medical College, Jodhpur (Reference No. SNMC/IEC/2023/2177-2178). Written informed consent was obtained from all participants prior to enrolment. Confidentiality of participant data was maintained throughout the study.

Statistical analysis

All data were recorded, compiled, and analyzed using SPSS version 26.0 (IBM Corp., Armonk, NY, USA). Continuous variables are expressed as mean±standard deviation (SD). Categorical variables are expressed as frequencies and percentages. Differences in continuous variables between two independent groups were assessed using the unpaired Student's t test. Categorical variables were compared using the Chi-square test; Fisher's exact test was applied where expected cell counts were less than five. Receiver operating characteristic (ROC) curve analysis was performed to determine the optimal serum homocysteine cutoff for predicting young-onset ACS (<40 years), and the area under the curve (AUC) with its 95% confidence interval (CI) was calculated. The Youden index (sensitivity+specificity-1) was used to identify the optimal cutoff point. A p-value <0.05 was considered statistically significant for all analyses.

## Results

A total of 75 patients with confirmed ACS were enrolled, with a mean age of 53.3±13.3 years (range 22-72 years) and a male predominance of 84% (n=63). STEMI accounted for the largest proportion of ACS subtypes (46.7%), followed by NSTEMI and unstable angina in equal measure (26.7% each). Sixteen patients (21.3%) were classified as having young-onset ACS (aged below 40 years), while the remaining 59 (78.7%) were aged 40 years or above. Baseline demographic and clinical characteristics are summarized in Table 1. The two age groups were well matched for ACS subtype distribution, prevalence of diabetes mellitus, hypertension, and smoking history, indicating that observed differences in study variables are unlikely to reflect disparities in conventional risk factor burden or ACS severity. In contrast, dyslipidemia and a family history of premature coronary artery disease were significantly more prevalent among younger patients, who also exhibited a more adverse lipid profile, suggesting that non-conventional and hereditary risk factors may assume greater pathogenic relevance in this subgroup.

**Table 1:** Baseline demographic and clinical characteristics stratified by age group ^*^Unpaired t test; ^#^Fischer exact test;^ $^Chi-Square test. LDL, low-density lipoprotein; HDL, high-density lipoprotein; ACS, acute coronary syndrome; STEMI, ST-segment elevation myocardial infarction; NSTEMI, non-ST-segment elevation myocardial infarction; CAD, coronary artery disease; SD, standard deviation

Parameter	<40 years (n=16, n(%))	≥40 years (n=59, n(%))	t/χ²	P-value
Age, mean±SD (years)	31.8±6.5	59.2±7.2	t=-13.77	<0.0001^*^
Sex: male	16 (100%)	47 (79.7%)	Fisher's exact	0.113^#^
Sex: female	0 (0%)	12 (20.3%)	Fisher's exact	0.113^#^
BMI, mean±SD (kg/m²)	26.9±2.0	27.7±1.5	t=-1.76	0.089^*^
ACS type				
STEMI	7 (43.8%)	28 (47.5%)	χ²=0.07	1.000^#^
NSTEMI	5 (31.2%)	15 (25.4%)	χ²=0.22	0.752^#^
Unstable angina	4 (25.0%)	16 (27.1%)	χ²=0.03	1.000^#^
Conventional risk factors				
Diabetes mellitus	8 (50.0%)	30 (50.8%)	χ²=0.004	1.000^$^
Smoking	11 (68.8%)	24 (40.7%)	χ²=3.99	0.087^$^
Hypertension	8 (50.0%)	26 (44.1%)	χ²=0.18	0.889^$^
Dyslipidemia	14 (87.5%)	17 (28.8%)	χ²=17.88	0.0001^$^
Family history of premature CAD	11 (68.8%)	17 (28.8%)	χ²=8.58	0.008^$^
Lipid profile				
Total cholesterol (mg/dL)	228.1±43.5	185.3±52.8	t=2.98	0.004^*^
LDL (mg/dL)	149.4±25.5	115.0±44.1	t=2.98	0.004^*^
HDL (mg/dL)	35.6±5.6	40.7±7.4	t=-2.56	0.011^*^
Triglycerides (mg/dL)	173.4±18.1	147.4±19.7	t=4.76	0.0001^*^

**Figure 1:**
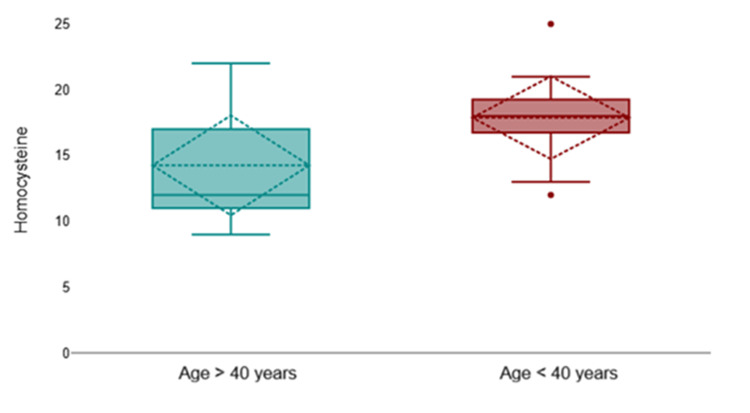
Box-and-whisker plot comparing serum homocysteine levels (µmol/L) between ACS patients aged below 40 years and those aged 40 years and above. The dashed diamond overlay represents the 95% CI around the median ACS, acute coronary syndrome; CI, confidence interval

Serum homocysteine levels were significantly higher in younger patients than in their older counterparts (17.9±3.2 vs 14.3±3.8 µmol/L; p=0.0009), with an overall cohort mean of 15.0±4.0 µmol/L. These findings are detailed in Table 2 and illustrated in Figure 1.

**Table 2:** Serum homocysteine levels according to age category and Lp(a) status ^*^Unpaired t test; ^#^Fischer exact test Lp(a), lipoprotein(a); SD, standard deviation

Parameter	<40 years (n=16, n(%))	≥40 years (n=59, n(%))	t/χ²	P-value
Homocysteine, mean±SD (µmol/L)	17.9±3.2	14.3±3.8	t=3.47	0.0009^*^
Elevated Lp(a) ≥30 mg/dL	14 (87.5%)	21 (35.6%)	χ²=13.63	0.0007^#^
Vitamin B12, mean±SD (pg/mL)	209.9±173.7	462.9±308.9	t=-3.13	0.003^*^
B12 deficiency (<200 pg/mL)	13 (81.2%)	26 (44.1%)	χ²=6.97	0.018^#^

Elevated Lp(a) was similarly disproportionate in the younger age group, affecting 87.5% of patients below 40 years versus 35.6% of those aged 40 years and above (p=0.0007), reflecting a clustering of non-conventional risk factors in young-onset ACS. When the contemporary guideline threshold of ≥50 mg/dL was applied to the dataset, only one patient (1.3%) met the criteria for elevated Lp(a), precluding meaningful subgroup analysis at this cutoff. The ≥30 mg/dL threshold was therefore retained as the operational definition throughout.

Vitamin B12 deficiency was identified in 52.0% of the overall cohort and was significantly more prevalent among younger patients (81.2% vs 44.1%; p=0.018), with mean serum B12 levels also significantly lower in this subgroup (Table 2). This pattern suggests that nutritional deficiency may be a contributing driver of hyperhomocysteinaemia in young-onset disease.

**Figure 2:**
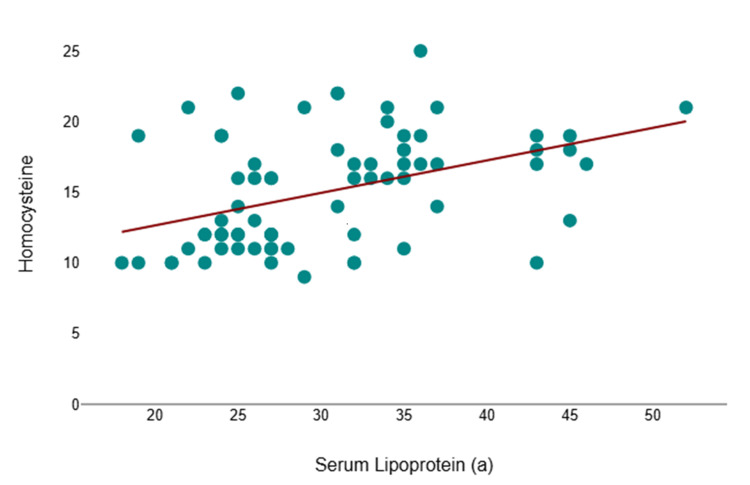
Scatter plot depicting the correlation between serum Lp(a) (mg/dL) and serum homocysteine (µmol/L) in ACS patients (n=75) ACS, acute coronary syndrome; Lp(a), lipoprotein(a)

When stratified by Lp(a) status, patients with elevated Lp(a) demonstrated markedly higher homocysteine concentrations than those with normal Lp(a) levels (17.0±3.6 vs 13.3±3.5 µmol/L; p<0.0001). This strong positive association, summarized in Table 3 and illustrated in Figure 2, suggests a potential synergistic interplay between hyperhomocysteinaemia and elevated Lp(a) in amplifying atherosclerotic risk, particularly among younger patients with ACS.

**Table 3:** Serum homocysteine levels according to Lp(a) status ^*^Unpaired t test Lp(a), lipoprotein(a); SD, standard deviation

Parameter	Elevated Lp(a) (n=35)	Normal Lp(a) (n=40)	Total (n=75)	t/χ²	P-value
Homocysteine, mean±SD (µmol/L)	17.0±3.6	13.3±3.5	15.0±4.0	t=4.51	<0.0001^*^

ROC curve analysis was performed to evaluate the utility of serum homocysteine as a discriminator of young-onset ACS (<40 years) within the study cohort. The AUC was 0.768 (95% CI 0.623-0.913; p=0.0005), indicating good discriminatory ability (Table 4).

**Table 4:** ROC curve analysis of serum homocysteine for predicting young-onset ACS ROC, receiver operating characteristic; ACS, acute coronary syndrome; AUC, area under the curve; CI, confidence interval

Parameter	Value
AUC (95% CI)	0.768 (0.623-0.913)
P-value	0.0005
Optimal cutoff	>17 µmol/L
Sensitivity	75.0%
Specificity	69.5%
Positive predictive value	40.0%
Negative predictive value	91.1%
Youden index	0.445

**Figure 3:**
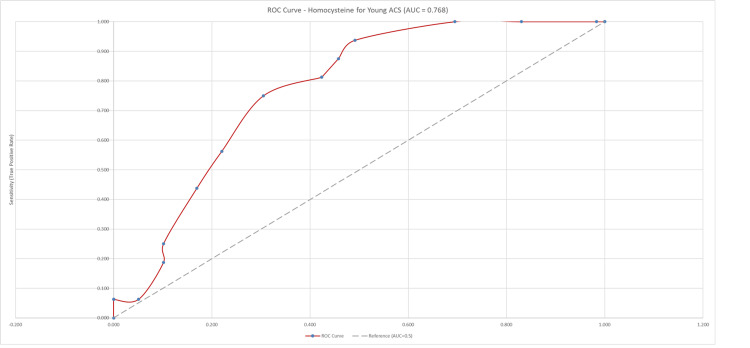
ROC curve for serum homocysteine as a predictor of young-onset ACS (<40 years). The diagonal reference line represents chance discrimination (AUC=0.50) ROC, receiver operating characteristic; ACS, acute coronary syndrome; AUC, area under the curve

At an optimal cutoff of >17 µmol/L, serum homocysteine distinguished young-onset from later-onset ACS with a sensitivity of 75.0%, specificity of 69.5%, positive predictive value of 40.0%, and negative predictive value of 91.1% (Youden index 0.445), as illustrated in Figure 3. Given that this threshold was derived from only 16 patients in the younger subgroup, it should be interpreted as exploratory and requires prospective validation in a larger, independent cohort before clinical application can be considered.

## Discussion

The present cross-sectional study evaluated serum homocysteine and serum Lp(a) levels in 75 ACS patients at a tertiary care center in Jodhpur, Rajasthan, with particular focus on age-specific differences and the interrelationship between these two non-conventional cardiovascular risk factors. The findings demonstrate that both hyperhomocysteinaemia and elevated Lp(a) are significantly more prevalent in younger ACS patients (<40 years), that elevated Lp(a) is strongly associated with higher homocysteine levels, and that serum homocysteine demonstrates good discriminatory ability for predicting young-onset ACS, with an AUC of 0.768.

The mean serum homocysteine level in patients aged <40 years was 17.9±3.2 µmol/L, significantly higher than the 14.3±3.8 µmol/L observed in those aged ≥40 years (p=0.0009). This is consistent with Shih et al., who demonstrated a significant association between elevated homocysteine and CVD risk in middle-aged and elderly adults in Taiwan, and with Meena et al., who reported significantly elevated homocysteine levels in Indian CAD patients compared with age-matched controls, confirming its role as an independent risk factor [16,17]. The finding of a stronger homocysteine signal in younger patients aligns with the established observation that non-conventional risk factors assume proportionally greater importance in young-onset CAD, where the accumulation of conventional risk factors is lower [3]. Unadkat et al. further demonstrated in a comprehensive systematic review and meta-analysis that the homocysteine-CAD association is stronger in Asian populations than in Western counterparts and has strengthened over time, lending important regional context to our findings [18].

A critical mechanistic explanation for the age-specific homocysteine finding lies in the vitamin B12 data from the present study. B12 deficiency (<200 pg/mL) was present in 81.2% of patients aged <40 years compared to 44.1% of older patients (p=0.018), with mean B12 levels significantly lower in the younger group. Since vitamin B12 is an essential cofactor for remethylation of homocysteine to methionine via methionine synthase, its deficiency directly impairs homocysteine clearance [4]. The resulting accumulation of homocysteine thiolactone thiolates LDL particles, facilitating macrophage endocytosis and foam cell formation, thereby potentiating atherogenesis [5-12]. Dietary patterns in younger urban Indians, characterized by declining intake of B12-rich animal products, may contribute to this deficiency and represent a readily modifiable upstream determinant of hyperhomocysteinaemia. Routine screening for vitamin B12 deficiency in young ACS patients may identify a readily modifiable upstream determinant of hyperhomocysteinaemia; however, as the present study did not evaluate clinical outcomes following supplementation, whether correction of B12 deficiency translates into reduction of homocysteine levels or cardiovascular risk in this population remains speculative and warrants prospective interventional investigation.

Elevated Lp(a) (≥30 mg/dL) was found in 87.5% of patients aged <40 years compared to 35.6% of older patients (p=0.0007), the most striking between-group difference observed in the dataset. This is consistent with the understanding that Lp(a) is genetically determined and its relative contribution to early-onset atherosclerosis is proportionally greater in younger individuals in whom acquired risk factors have had less time to accumulate [13]. The inflammatory and pro-atherogenic burden of Lp(a), alongside other emerging risk factors, has been well characterized by Blake and Ridker, who demonstrated that such biomarkers provide significant predictive value beyond conventional risk stratification [14]. A significant positive association was further found between elevated Lp(a) and higher homocysteine across the entire cohort (17.0±3.6 vs 13.3±3.5 µmol/L; p<0.0001), with a significant positive correlation on continuous analysis (Pearson r=0.413; p=0.0002), corroborated by Foody et al., who found that co-elevation of these two biomarkers conferred a cardiovascular risk greater than the sum of their independent effects, particularly in women [15]. Mechanistically, homocysteine promotes oxidative modification of Lp(a) particles and impairs fibrinolysis through plasminogen competition, creating a synergistically pro-thrombotic milieu; endothelial injury arising from these pathways may further impair coronary microvascular function, contributing to myocardial ischaemia through mechanisms beyond epicardial atherosclerosis, as reflected in abnormal myocardial perfusion kinetics observed in high-risk patient profiles [5-12,19].

ROC curve analysis demonstrated that serum homocysteine at a cutoff of >17 µmol/L predicted young-onset ACS with a sensitivity of 75.0%, specificity of 69.5%, and a high negative predictive value of 91.1% (AUC 0.768, 95% CI 0.623-0.913; p=0.0005).

The stratified baseline data revealed additional noteworthy findings. Dyslipidemia (87.5% vs 28.8%, p=0.0001), family history of premature CAD (68.8% vs 28.8%, p=0.008), and a significantly more deranged lipid profile were all more prevalent in the younger group, while ACS subtype distribution was comparable between groups (p=0.896), confirming that the observed differences in homocysteine and Lp(a) are unlikely to be attributable to differing ACS severity. Taken together, young-onset ACS in this cohort is characterised by a convergence of genetic predisposition, metabolic dysregulation, and nutritional deficiency. The response-to-injury hypothesis contextualises this coherently: homocysteine, alongside haemodynamic stress, hyperlipidaemia, and inflammatory cytokines such as tumour necrosis factor, acts as a convergent trigger for endothelial injury, initiating the atheromatous cascade [1].

The study has some limitations. Being a single-center cross-sectional study with 75 patients, generalizability is limited, and causal inference cannot be drawn. A healthy age-matched control group was not included. Serum folate levels and dietary assessment data were not recorded. Application of the contemporary ≥50 mg/dL Lp(a) threshold yielded only one eligible patient in this cohort, precluding its use as an analytical cutoff; future studies in Indian ACS populations are needed to determine an appropriate cutoff. The ROC-derived homocysteine cutoff of >17 µmol/L is based on a subgroup of 16 patients and should be regarded as hypothesis-generating; external validation in adequately powered prospective studies is necessary before this threshold can inform clinical practice. Methylenetetrahydrofolate reductase (MTHFR) genotyping, which would have allowed identification of individuals with genetically impaired homocysteine remethylation, was not performed owing to financial constraints and the unavailability of the test at our institution. Future multicenter prospective studies with control groups and genetic profiling are warranted to validate these findings.

## Conclusions

The present study establishes that serum homocysteine and serum Lp(a) are significantly elevated in ACS patients younger than 40 years, and that a strong positive association exists between these two non-conventional risk factors, suggesting a synergistic role in accelerating atherogenesis in young-onset CAD. The high prevalence of vitamin B12 deficiency in the younger cohort identifies a readily modifiable and often overlooked upstream determinant of hyperhomocysteinaemia. Given the cross-sectional design, small sample size, and absence of a control group, these findings are best regarded as hypothesis-generating. Estimation of serum homocysteine, Lp(a), and vitamin B12 may warrant consideration in the workup of younger ACS patients in Indian clinical practice, particularly as inexpensive and widely available investigations; however, prospective multicenter studies with adequate controls are necessary before definitive incorporation into the standard biochemical workup can be recommended.
